# Combined Approach for Giant Temporal Meningoencephalocele

**DOI:** 10.22038/IJORL.2022.66306.3266

**Published:** 2023-01

**Authors:** Claudio Carnevale, Miguel Garcia-Wagner, Carolina Morales-Olavarría, Pedro Sarría-Echegaray, Guillermo Til-Pérez

**Affiliations:** 1 *Department of ENT and Head and Neck, Hospital University Son Espases, Palma de Mallorca, Spain.*; 2 *Department of ENT and Head and Neck, Rotger Clinic, Palma de Mallorca, Spain.*

**Keywords:** Acquired Encephalocele, Cholesteatoma, Middle ear, Temporal bone, Skull base

## Abstract

**Introduction:**

To present a complex case of giant meningoencephalocele after a canal wall down mastoidectomy and describe our preferred approach to repair meningoencephalic herniation of the temporal bone.

**Case Report:**

A 20-year-old patient, who had previously undergone type III tympanoplasty with total ossicular reconstruction prosthesis for an attic cholesteatoma, presents with clinical and imaging features compatible with the diagnosis of a giant temporal meningoencephalocele. We performed a combined approach –transmastoid plus minicraniotomy- to repair the skull base defect. A multilayer reconstruction of the defect with septal cartilage and temporal fascia was performed. After a 48 months follow-up, the patient remains symptom free without signs of tissue herniation.

**Conclusions:**

Transmastoid plus minicraniotomy combined approach is a safe and feasible technique in case of large and anterior skull base defects with low surgical morbidity, allowing a safe and multilayered reconstruction, even in the context of a simultaneous active chronic otitis media.

## Introduction

Brain and meningeal tissue herniation through the tegmen tympani into the temporal bone rarely occurs (incidence 1/35,000), but it represents a potentially life-threatening condition that should always be surgically corrected. This entity is commonly known as a temporal encephalocele, although some authors believe ‘‘meningoencephalic herniation’’ (MEH) is a more appropriate term, given that a cystic area containing cerebrospinal fluid (CSF) is not a persistent finding (1). 

The existence of a skull base defect is an imperative condition for the development of an encephalocele, but the cause of the defect is variable. Chronic otitis media, with or without cholesteatoma, otologic surgery, temporal bone fracture, infections, tumors and congenital defect of skull base are all possible causes for a temporal encephalocele to develop; spontaneous encephaloceles represent approximately the 20% of the cases (2-4).

Regardless of the cause, the communication between the middle ear and the subarachnoid space could be a risk factor for cerebral infections and the presence of herniated dysfunctional cerebral tissue may trigger epilepsy. 

Clinical presentation is variable, common symptoms are headache, epileptic seizures, conductive hearing loss due to CSF fistula and recurrent meningitis It could also be diagnosed as an incidental finding on a computed tomography (CT) or magnetic resonance imaging (MRI). 

The analysis of beta-trace protein confirms the presence of a CSF fistula, while a CT scan and MRI identify the location and the size of the defect. We report a rare case of a giant temporal bone encephalocele after a canal wall down mastoidectomy and describe our favorite surgical technique for skull base defect repair. 

## Case Report

**Fig.1:**
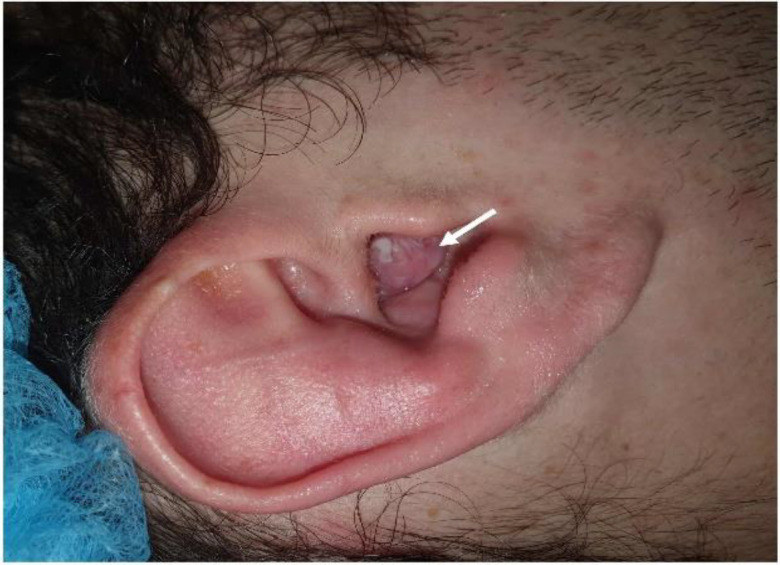
A mass protruding into the external auditory meatus is identified

We report the case of a 20-year-old patient who was referred to the ENT Department of our hospital due to a mass protruding into the external auditory meatus with a conductive hearing impairment (Fig. 1). 

One year before, the patient had undergone retrograde on-demand mastoidectomy for the treatment of an attic cholesteatoma. 

**Fig. 2:**
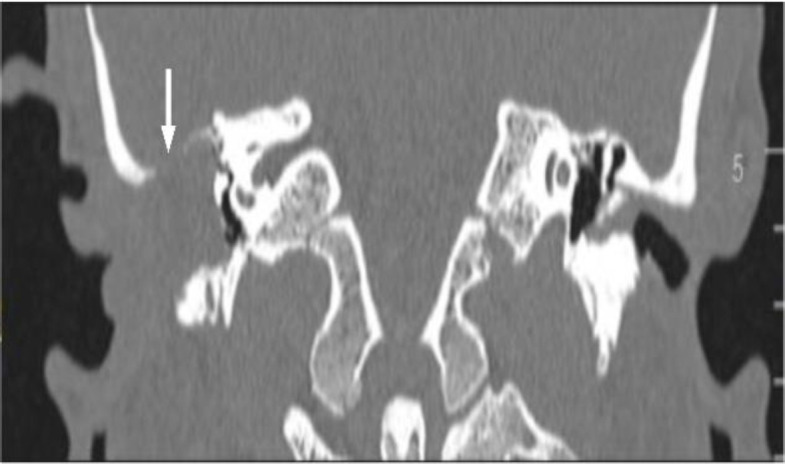
CT scan. Coronal view. A right skull base defect is observed

CT scan demonstrated the presence of a bony defect in right tegmen timpani of more than 1 cm width (Fig. 2).

**Fig.3:**
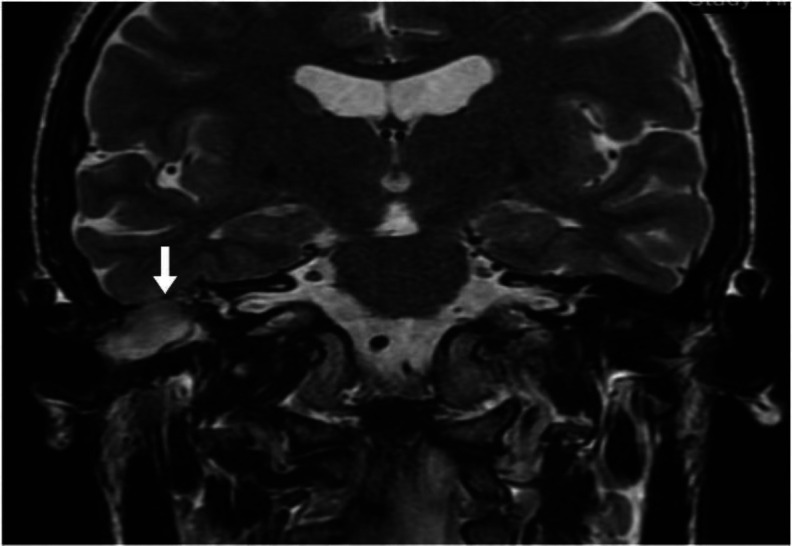
MRI imaging. Coronal view. Brain tissue herniation is identified on the right side

MRI showed a herniated tissue from the right inferior temporal lobe, which confirmed the diagnosis of temporal meningoencephalocele (Fig.3). Revision surgery was planned and a combined approach – transmastoid + minicraniotomy- performed. 

Under facial nerve monitoring a retroauricular incision was marked and the mastoid cavity performed during the previous mastoidectomy was reached. 

**Fig. 4:**
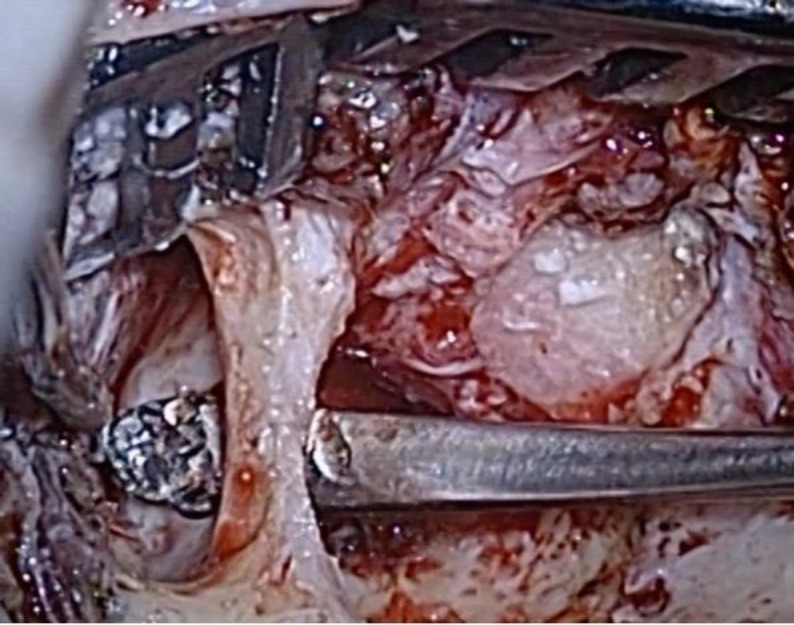
Dura is elevated from the middle cranial fossa floor in a lateral to medial direction and the bony defect is identified from above and from below

The devitalized herniated tissue was bipolar-coagulated and amputated, until the tegmen defect was identified. After harvesting a temporalis fascia graft, a 2 x 1.5 cm craniotomy was created by drilling approximately 2-3 mm above the temporal line. Using a freer elevator, the dura was elevated from the middle cranial fossa floor in a lateral to medial direction until the bony defect and its residual herniated tissue were exposed (Fig. 4). 

**Fig. 5:**
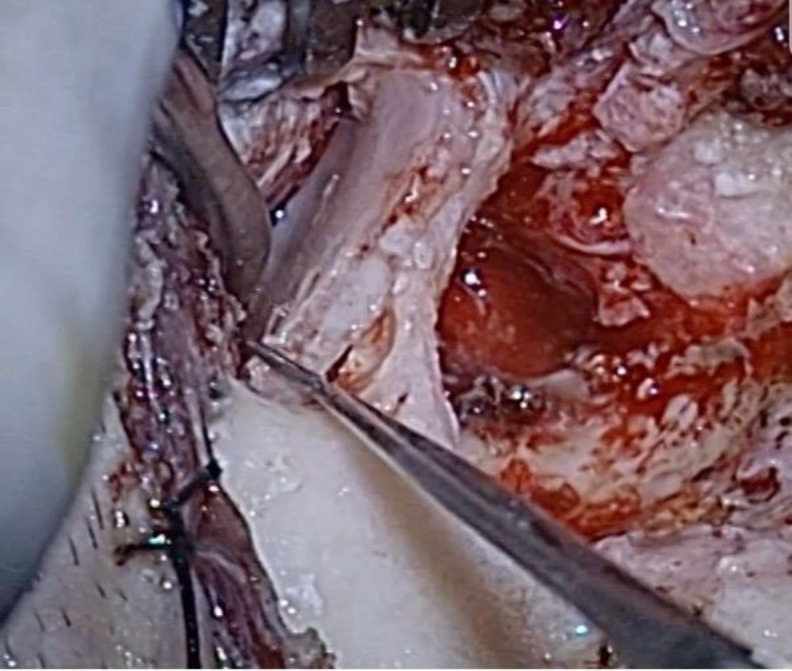
Septal cartilage graft is disposed between the dura and the floor of middle fossa, covering the bony defect

A previously harvested cartilage nasal septal graft was disposed between the dura and the floor of middle fossa, covering the bony defect with a significant support margin (Fig. 5). 

Then, several smaller cartilage grafts and the temporalis fascia graft were placed to seal the defect from the mastoid in a multilayer reconstruction and covered by fibrin glue (Tissucol). Postoperative course was uneventful, and the patient was discharged on the fourth postoperative day after performing a CT scan, which confirmed the absence of intracranial complications. After a 48 months follow-up, the patient remains symptom free, otomicroscopy and MRI show a complete seal of the tegmen timpani. 

## Discussion

Temporal encephalocele is a rare entity with multiple etiologies. Although the presence of a bony defect is considered necessary for the development of a brain herniation, postmortem anatomical findings suggest that encephalocele is not an inevitable sequela of tegmen dehiscence (5). Encephalocele may be classified into spontaneous and secondary to specific etiology. Different theories have been described to explain the pathogenesis of spontaneous herniations, such as variations of intracranial pressure, chronic CSF pulsation, low-grade inflammation, or aberrant arachnoid granulations (6,7). 

Among secondary encephaloceles, otologic surgery to treat chronic otitis media with or without cholesteatoma, represent the most common etiology. Several authors suggest that dural injury is a prerequisite for iatrogenic encephalocele (8). However, even without previous bony and/or dura injury, encephalocele herniation has been described in the literature (9). Surgical treatment is always required since the existence of a communication with the subarachnoid space may predispose to life-threatening complications. Four surgical approaches have been described: 1) transmastoid; 2) fossa media; 3) combined –trans-mastoid plus fossa media- and 4) mastoid and middle ear obliteration. The surgical approach depends on the etiology, the site, the size and the number of bony defects, the existence of associated middle-ear infection, preoperative hearing thresholds and the surgeon’s experience (7-10). Independently of the approach, a multilayered reconstruction is considered the best technique to avoid any relapse. Transmastoid approach is usually used for small bony dehiscence posteriorly located at the level of tegmen mastoideum or antri, with no need of manipulating the ossicular chain. In case of more anterior defects, the disarticulation and removal of the ossicular chain may be needed. Defects greater than 1 cm and multiple defects represent the principal contraindication for this approach, although exceptional cases with large defects have been successfully repaired. The middle fossa approach allows for total control of the middle fossa floor, reaching larger and anterior defects without manipulation of the ossicular chain. As described by the “Gruppo Otologico” (7), this technique is especially useful in case of spontaneous encephalocele without middle ear pathology. The need for intensive care unit and potential complications (temporal lobe injury, seizure, and hematoma) represent some of the disadvantages of this technique, especially in elderly patients. The mastoid and middle ear obliteration with closure of the eustachian tube is considered an extreme option since a subtotal petrosectomy is performed and moderate/severe conductive hearing loss is caused. It represents a definitive solution for chronically infected ears, with no possibilities of hearing reconstruction (10). 

Combined trans-mastoid plus minicraniotomy approach offers a bilateral control of the herniated tissue from above and below. With this approach it is possible to treat middle ear pathology, reconstruct the ossicular chain and repair the tegmen defect in the same surgery. Compared with the transmastoid approach, it allows for a better control of anterior and/or large defects. With respect to the middle fossa approach, less manipulation of the temporal lobe is needed. However, both a post-operative CT scan and 24 hours in the ICU are recommended. 

## Conclusion

The authors consider that the combined approach is a safe and feasible technique to repair anterior and large or multiple tegmen defects without adding significant morbidity. 

It is also the first option in case of a simultaneous chronic ear infection allowing the repairing of the ossicular chain in the same surgical act.
